# How can you capture cultural dynamics?

**DOI:** 10.3389/fpsyg.2014.00995

**Published:** 2014-09-10

**Authors:** Yoshihisa Kashima

**Affiliations:** Melbourne School of Psychological Sciences, The University of MelbourneParkville, VIC Australia

**Keywords:** cultural dynamics, culture change, cultural evolution, history, methodology

## Abstract

Cross-cultural comparison is a critical method by which we can examine the interaction between culture and psychological processes. However, comparative methods tend to overlook cultural dynamics – the formation, maintenance, and transformation of cultures over time. The present article gives a brief overview of four different types of research designs that have been used to examine cultural dynamics in the literature: (1) cross-temporal methods that trace medium- to long-term changes in a culture; (2) cross-generational methods that explore medium-term implications of cultural transmission; (3) experimental simulation methods that investigate micro-level mechanisms of cultural dynamics; and (4) formal models and computer simulation methods often used to investigate long-term and macro-level implications of micro-level mechanisms. These methods differ in terms of level of analysis for which they are designed (micro vs. macro-level), scale of time for which they are typically used (short-, medium-, or long-term), and direction of inference (deductive vs. empirical method) that they imply. The paper describes examples of these methods, discuss their strengths and weaknesses, and point to their complementarity in inquiries about cultural change. Because cultural dynamics research is about *meaning over time*, issues deriving from interpretation of meaning and temporal distance between researchers and objects of inquiry can pose threats to the validity of the research and its findings. The methodological question about hermeneutic circle is recalled and further inquiries are encouraged.

## HOW CAN YOU CAPTURE CULTURAL DYNAMICS?

In the early 21st century world, the cultures that we used to take for granted as part of the immutable reality seem to be changing. Call them globalization or culture change, whatever drives the shared impression of the changing world, the dynamic movement of culture is a critical question facing humanity today. Whereas the classics of social science were born out of the observation of cultural changes in the 19th and early 20th century (often called modernization or industrialization), social science is again spurred by the contemporary experience of changing life. There are, however, critical theoretical and methodological questions – how can we capture the contemporary cultural dynamics?

Although the traditional social science has used a comparative historical analysis (Mahoney and Rueschemeyer, 2003) to postulate and substantiate the theories of cultural change, contemporary research has developed various methods that were unavailable a century ago, which may enable us to consider and examine novel theoretical ideas, uncover new findings, and bring forward new insights. The purpose of this article is to take stock of those methods that have recently emerged. It does not exhaustively catalog the research that makes use of these methods, but categorize different methods, characterize them in general terms, and discuss their strengths and limitations, so as to aid principled examination of cultural dynamics.

## WHAT IS CULTURE AND CULTURAL CHANGE?

Before discussing the method of examining cultural change, it is necessary to clarify what is meant by cultural change. An answer rests on the difficult question of what is meant by culture. Although there has been a number of definitions of the culture concept (e.g., Baldwin et al., 2006, for a recent survey), the one adopted here is arguably an outgrowth of the cognitive revolution. It regards cultural information as non-genetic information that is transmissible from one person to another and that can potentially affect the person’s behavior (e.g., Kashima, 2000a, 2008, 2014). To the extent that given cultural information is widespread in a given group of people, it constitutes part of the group’s culture. A culture, then, is a set of non-genetic information that is available (i.e., information exists), accessible (i.e., information can be acquired), and applicable (i.e., information is usable) to a group of people.

This understanding of culture is not unique. A number of psychologists (e.g., Campbell, 1975; Hong et al., 2000; Chiu and Hong, 2006), cognitive scientists (e.g., Sperber, 1996), anthropologists (e.g., Cavalli-Sforza and Feldman, 1981; Boyd and Richerson, 1985), and biologists (e.g., Dawkins, 1976) have adopted a similar conceptualization. Thus, a cultural change is defined as a *change in the set of non-genetic information available, accessible, and applicable in a group*. Here is one caveat. It is possible to think of the whole of humanity (or *Homo sapiens sapiens*) as a group and conceptualize a change in the distribution (not only frequency, but also geographical distribution) of scores on a measure defined over the set. The purpose of an investigation determines what the group and its culture is. Cultural dynamics, then, is an investigation of how a culture thus defined is formed, maintained, and transformed over time.

## PHENOMENA OF CULTURAL DYNAMICS

There are at least four basic sources of cultural dynamics.

• *Importation*: new cultural information that has not existed in a given culture, but which has existed in a different culture, is added to the former culture by virtue of transmission from the latter.
• *Invention*: new cultural information that has not existed in the culture of a group is added to the culture without importation.
• *Selection*: cultural information is selected *in* for further reproduction or selected *out* to be removed from a culture.
• *Drift*: random processes produce a change in prevalence of cultural information over time.

Invention and importation are concerned with the *addition* of information to a culture, whereas selection and drift can change the *prevalence* (or frequency of occurrence) of cultural information upwards or downwards, and in some cases potentially cause a loss (subtraction or removal) of cultural information as well. Considered this way, there are two general classes of questions about cultural dynamics. First is about *what*, that is, descriptive cultural dynamics – what cultural information exists, how prevalent it is, and how its existence and prevalence has changed and will change over time. The second class of questions is about *how*, namely, the mechanisms of cultural dynamics – how a change occurs by what mechanisms. These questions also tend to differ in *level of analysis.* Typically, descriptive cultural dynamics are concerned with *macro-level trends* of appearance and disappearance of cultural information, as well as increasing, decreasing, or steady state trajectories of cultural information prevalence in a group. *Micro-level mechanisms* of cultural dynamics typically provide explanations for the macro-level trajectories of cultural change. The level of analysis is often, though not always, aligned with *time scale*. The macro-level trend questions are typically concerned with a longer-term time scale, whereas the micro-level mechanism questions are not so concerned about long-term, but can often be answered in short-term.

### MACRO-LEVEL CULTURAL DYNAMICS

The theory of Western modernization since Tönnies (1955), Weber (1958), and Durkheim (1964) as well as the contemporary research on individualism and collectivism, has an often tacit assumption that a culture has changed *unidimensionally* from a collectivist past to contemporary individualism (e.g., Triandis, 1989). However, there is a prominent *multidimensional* model of cultural change. Inglehart’s (1977, 1997) and Inglehart and Baker (2000) theorizing about materialism and postmaterialism highlights a degree of discontinuity in the direction of cultural dynamics. According to him, *materialist* values emphasize hard work, money, and economic security, whereas *postmaterialist* values include self-expression, generalized trust, and environmental protection. As a society becomes more aﬄuent, it moves from a materialist to postmaterialist orientation. Whereas he acknowledges a degree of continuity from materialism to postmaterialism in terms of secularization (i.e., less emphasis on religion) and individuation (i.e., individualism), and therefore, does not reject the classical collectivism-to-individualism thesis, he suggests that postmaterialism is not continuous with materialism.

In addition to the unidimensionality vs. multidimensionality of cultural change, macro-level cultural changes differ in *trajectory*. One trajectory is *gradualism*, according to which a culture changes gradually over time; the other is *punctuated equilibrium*, which says that a culture changes in fits and starts, so that there are periods of stability over time, which are punctuated by rapid changes. Gradualism is analogous to what is typically regarded as the Darwinian evolution – a small amount of change is cumulated one at a time, and the cumulative effects amount to a visible and large change over a long period of time. Punctuated equilibrium is a pattern of biological evolution Eldredge and Gould (1972) suggested (also see Gould and Eldredge, 1993), which was then adopted by social scientists (e.g., Kuhn, 1970; Gersick, 1988; Baumgartner and Jones, 1993; Dixon, 1997).

### MICRO-LEVEL CULTURAL DYNAMICS

Micro-level cultural dynamics are often discussed in terms of contributions to *cultural change* or *maintenance* (e.g., Kashima, 2008). If information consistent with prevalent cultural information is added to the culture of a group, it *maintains* the culture. In contrast, if information that is inconsistent with the prevalent cultural information or new cultural information that has not existed in a culture is added to the culture of a group, it can *change* the culture. Considered this way, many research questions about micro-level cultural dynamics would be concerned with the mechanisms that facilitate or inhibit the addition of culturally consistent, inconsistent, or novel information to a culture. The questions about the mechanisms of cultural maintenance and change – cultural invention, importation, drift, and selection – are primarily concerned with explanatory cultural dynamics, that is, explanations about why and how cultures remain stable and change over time.

Other research is critically concerned about factors that facilitate or inhibit the transmission and acquisition of cultural information. Boyd and Richerson (1985; also see Henrich and McElreath, 2003) classified them into direct, indirect, and frequency-dependent biases. *Direct biases* derive from the cost and benefit of given cultural information. If it is less costly or more beneficial for an individual to transmit, acquire, and use given information, it is more likely retained in a culture. For instance, if a cultural learner tries out a certain way of making an arrow and finds it beneficial for hunting, he or she is likely to adopt it. An intriguing example of direct bias is the possibility that human minds have an evolved bias to acquire religious ideas such as belief in supernatural beings (e.g., spirits, other disembodied beings with minds; Boyer, 1994; also see Norenzayan et al., 2006). This is because humans have an evolved tendency to believe in the existence of minds as separate from bodies (e.g., see Bloom, 2004). *Indirect biases* derive from the use of a characteristic of the transmitter as a basis for acquiring and using given cultural information. A cultural learner is more likely to imitate a person who is more successful and more prestigious (for a review, see Henrich and Gil-White, 2001). Also there is a tendency for a cultural learner to acquire cultural information from a similar others, especially his or her in group (e.g., Abrams et al., 1990). *Frequency-dependent biases* derive from the use of frequency information as a basis for cultural acquisition and use. So, a cultural learner tends to acquire and use information when it is used by a majority of people in the group (e.g., Asch, 1955; Cialdini et al., 1990; see Kashima et al., 2013).

## METHODOLOGY FOR CULTURAL DYNAMICS

Cultural dynamics research is an iterative process that starts with questions about the trajectory and mechanisms of cultural change and ends with a tentative conclusion about them. Researchers select a general research design which tends to constrain the choice of observation methods that enable the researchers to construct measurements. They are then converted to variables, statistical analyses are typically conducted, and conclusions are drawn. These conclusions are nonetheless tentative, giving rise to further research questions, prompting the research cycle to start yet again. As many thinkers of research methodology noted (e.g., Campbell and Stanley, 1963; Runkel and McGrath, 1972; Webb et al., 1981), it is a potentially endless iterative movement between the world of theoretical constructs and the social world of interest, whose outcome is hoped to increasingly better approximate the object of inquiry.

### RESEARCH DESIGN

Once a research question is identified, one of the first steps is to choose a research design (e.g., Runkel and McGrath, 1972). It is a set of mutually constraining research practices for an inquiry.

#### Cross-temporal method

The first class of research designs may be called *cross-temporal method*, in which data are collated over a period of time and the trajectory of the time-stamped data points is examined. Just as cultural psychology’s prototypical research design is cross-cultural comparisons, cultural dynamics requires cross-temporal method whose modus operandi are comparisons across time.

***Archival research***. Archival research is perhaps the most prototypical design for cross-temporal method. An intriguing example of its use comes from Wolff et al. (1999), in which they examined the trajectory of biological knowledge in English culture over time. To do so, the authors traced the appearance of English words for trees (e.g., alder, ash, birch, maple, oak, pine) used in quotations included in the Oxford English Dictionary. They reviewed the original editor of the OED, James Murray’s method to gage the representativeness of the quotations collected in the dictionary, and then went about searching through its voluminous content by using a search engine (keeping in mind multiple spellings of the same word such as *trau*, *traw*, *tre*, etc. for *tree*), extracting relevant quotations, coding the entries (e.g., excluding the use of a tree term in a proper name such as “Sir Mulberry Hawk”), and correcting for biases in the sampling of quotations in the OED (e.g., sampling of quotations for some period was greater than in other periods, and so the counts were normalized against the estimated total number of quotations in each period). Their analysis showed that the appearance of tree terms was generally steady from the 16th to 18th century, showed a steep increase to the 19th century, and showed a sharp decline to the first half of the 20th century, generally showing both an evolution and devolution of folk biological knowledge.

Archival data are more often obtained from what (Webb et al., 1981; also Lee, 2000) called *running records*. They typically come from bureaucratic records that are kept for purposes other than research. For instance, Oishi et al. (2013) examined a historical trend in the use of the term “happiness” in the State of the Union Addresses in the US. They identified two senses in which the concept of “happiness” has been used: one is an older usage that refers to the prosperity of a collective as in “happiness of the man and that of his family,” and the other refers to a positive emotional state of an individual. They examined the use of the term “happy” or “happiness” in the first sense, and found that earlier presidents were more likely to use the term in the sense of luck, fortune, and prosperity of a country or a collective than more recent presidents, noting that President Reagan was the only president over the past 30 years to use *happy* in this old-fashioned way. The suggestion is that the collectivistic usage of the term has declined over time at least in the US public discourse. There are other intriguing use of running records such as baby names (e.g., Berger and Le Mens, 2009; Berger et al., 2012).

There are other archival data, which Webb et al. (1981) called *episodic* or *private records*, which were not kept as periodical recording of events and state of affairs. They include diaries, personal letters, and other occasional publications that are publically available. These may have been almost exclusively a historians’ data source, but more recently, a highly useful resource for this type of archival data has become available through Google Books. Michel et al. (2011) reports their construction of a corpus of more than five million digitized books (or approximately 4% of all published books). It contains more than 500 billion words in English, French, Spanish, German, Chinese, Russian, and Hebrew. The search engine associated with this data base called Google Ngram Viewer enables users to obtain the frequency of usage of a 1-g (i.e., a string of characters uninterrupted by a space, including words such as “banana”) up to 5-g (i.e., a string of characters interrupted by a space four times, such as “the United States of America”). Frequency counts are normalized for the number of words in the corpus for each year.

Google Ngram has been used to investigate macro-level cultural trends in the United States. For example, Twenge et al. (2012b, 2013) found the use of individualistic concepts (e.g., independent, individual, unique) and pronouns (e.g., I, my, me) showed an increasing trend in the US from 1960 to 2008. Conversely, Kesebir and Kesebir (2012) found that the use of moral virtue words (e.g., discipline, honesty, courage, wisdom) largely declined over the 20th century (1901–2000), suggesting a general decline in virtue ethics, arguably a correlate of collectivism. Generally in line with this set of findings, Greenfield (2013) examined the usage of English words that appear to signify a community-based lifestyle (Gemeinschaft; “obliged,” “give,” and “act”) as opposed to a more urban lifestyle (Gesellschaft; “choose,” “get,” and “feel”). Mirroring the population distribution in the rural vs. urban areas of the US, which showed a declining rural and a rising urban populations, the Gemeinschaft-like words showed a general decline, whereas the Gesellschaft-like words showed a general increase.

Michel et al. coined the term, *culturomics*, to capture this type of research in which “the application of high-throughput data collection and analysis to the study of human culture (Michel et al., 2011, p. 181).” It remains to be seen whether this neologism sticks or not.

***Cross-temporal meta-analysis***. Archival data are records of social behavior from the past, but they were not collected and kept for the purpose of research. In contrast, the past social scientific research itself can provide a record of the past social behavior. Research reports (e.g., published or unpublished papers) can then be used to obtain information to conduct a meta-analysis. Twenge pioneered this research method in her 1997 research on historical trajectories of attitudes toward women. Since then, she and her colleagues have conducted a series of cross-temporal meta-analyses on psychological measures including anxiety (Twenge, 2000), personality (e.g., Twenge, 2001; Twenge et al., 2004), self-esteem (e.g., Twenge and Campbell, 2001), and Narcissism (Twenge et al., 2008). Twenge (2011) gives a summary of this method. A researcher selects a construct of interest, identify its psychometrically sound measures, search the relevant data base for papers including PhD dissertations that cite the original paper that reports the measure of interest, extract relevant information including the mean levels of given birth cohorts at specified age (i.e., those who were born in a specified period of time), and examine the trend of the mean levels over time (year in which the research was conducted or published). A correlation between mean level and year is often computed to obtain information about a linear trend, and this statistic can be used to aggregate across different measures of the same construct, examine the effect size, and so on.

Despite these strengths, as Trzesniewski et al. (2008; also Trzesniewski and Donnellan, 2010) noted, the generalizability of the cross-temporal meta-analyses depends on the representativeness of the original research that they use. If the original studies used convenience samples, the generalizability of their findings to the whole of a birth cohort is limited. Still, the utility of cross-temporal meta-analysis should not be written off. Sometimes this may be the only method to look into the psychological constructs measured in the past.

***Cross-temporal analysis of running surveys***. There are a number of surveys that have been conducted repeatedly over a considerable period of time. Many examples come from the USA (e.g., World Values Surveys^1^; for high school seniors, Monitoring the Future^2^; for college students, The American Freshman^3^) and Europe (e.g., Eurobarometer^4^), but there are others from Africa (e.g., Afrobarometer^5^), Arab (Arab Barometer^6^), Asia (e.g., Asian Barometer^7^), and Latin America (AmericasBarometer^8^; Latinobarómetro^9^) as well. These are often publicly available data that have been collected over a number of years from defined cohorts of respondents. These data may be called *running surveys* because they are analogous to running records that are collected and kept for other purposes. They differ from running records, however, because they were collected and kept for the purpose of research designed and executed by other social scientists (e.g., political scientists, sociologists, education researchers) with their own research agendas.

There are a number of publicly available databases that enable cultural dynamics research. For example, two research groups, Trzesniewski et al. (2008), Twenge and Campbell (2008), Trzesniewski and Donnellan (2010), and Twenge et al. (2012a,b), have used several different data sets for American high school and university students to examine whether macro-trends towards greater individualism exist in the US. Using freshmen data from the University of California, Berkeley and Davis, Trzesniewski et al. (2008) reported little increase in positive self-regard, in particular, Narcissism as measured by the Narcissistic Personality Inventory (Raskin and Terry, 1988); using American high school seniors data, Twenge and Campbell (2008) reported an increase in self-liking, but not in self-competence. In a target article for *Perspectives on Psychological Science,* Trzesniewski and Donnellan (2010) used the high school seniors data that Twenge and Campbell (2008) used, and argued that there was little evidence to suggest trends towards greater positive self-regard on a range of items. Twenge and Campbell (2010) commented that although Trzesniewski and Donnellan (2010) regarded effect sizes of |*d*| <0.20 as trivial, many of the effects were highly statistically significant in the direction of greater positive self-regard.

Although both the research groups have their own perspectives and other researchers may evaluate them differently, their controversy highlights three important methodological issues. First, running surveys are not by definition designed to address the particular question that cultural dynamics researchers is interested in, and so they need to select relevant information from the available survey materials. In so doing, some researchers may select some items, whereas others may regard other items that the first researchers did not select. Here, clear conceptual definitions of a construct of interest would be essential. Second, some regard effect sizes need to be large enough for a macro-level trend to be theoretically meaningful; however, what effect sizes are meaningful enough becomes an important question in and of itself and statistical significance is one obvious way of settling the matter. Nonetheless, when the number of responses becomes very large (tens of thousands), the conventional type I error rate of 5% may be too lenient. Third, Roberts et al. (2010) noted that the effect size of cohort is not as great as the effect size of age, in that younger people are more narcissistic than older people. Their suggestion makes an important point that generational and developmental effects need to be carefully disentangled before conclusions about generational differences can be drawn (for statistical discussions surrounding this, see Yang and Land, 2008; O’Brien, 2011).

All in all, there is evidence to suggest that individualism is on the increase in the United States. However, in the Google Books research, there were some countervailing trends in these studies. Collectivistic phrases showed an increasing trend (Twenge et al., 2012b); first person plural (i.e., “we”) declined from the 60s to 80s, but then increased since then; and the use of words like “morality” and “ethics” increased in recent times (Kesebir and Kesebir, 2012). Likewise, Hamamura (2012) conducted cross-temporal analyses of running surveys in both the US and Japan, using indices and items available in both countries (e.g., World Values Survey, World Youth Survey), as well as those available only in the US (e.g., General Social Survey) and only in Japan (National Characteristic Survey). Hamamura selected those items that empirically correlated with international indices of individualism–collectivism, examined their macro-level trends over time, and found that some showed an increasing individualism and others showed an increasing collectivism in both countries. It was rather difficult to draw a clear conclusion either way in both the US and Japan.

One of the potential issues in this area of research is the conceptual definitions of individualism and collectivism. Although Hamamura used the items’ cross-national correlations with international indices to gage whether they tap individualism or collectivism, the Gen Me research does not appear to take this empirical approach to individualism. Whereas positive self-regard may be related to individualism across nations (e.g., Heine et al., 1999), there is evidence to suggest that it may be more related to economic inequality (Loughnan et al., 2011). The GenMe trend may be a cultural correlate of increasing inequality in the United States.

***Longitudinal research***. Some cross-temporal research is *longitudinal* in that researchers set out to study macro-level trajectories of cultural dynamics of a given group and design their research for the purpose. A well-known example is Inglehart’s World Values Survey (WVS). At the time of writing, the WVS project has collected five waves of data (1981–84, 89–93, 94–98, 99–04, 05–08) and the sixth wave is ongoing (due to end in 2014). Whether it was originally designed to be a longitudinal study, the team of researchers led by Ronald Inglehart developed a clear intent to capture values data over time from a large number of countries, each of which was to provide a national representative sample of at least 1000 survey respondents above 18 years of age. A survey questionnaire was developed in English, translated into the national language of the country in which data were collected (and sometime back-translated to English to check cross-cultural equivalence), and administered via face-to-face interviews.

Using the data up to 1998, Inglehart and Baker (2000) reported that their value items formed two distinct dimensions, traditional vs. secular-rational and survival vs. self-expression values, at both nation and individual levels. The traditional-secular dimension contrasts obedience and religious faith against independence and self-determination; the survival-expression dimension contrasts economic and physical security with self-expression and quality of life. Examining the historical trends in 38 countries, they reported that economic growth over time precipitated a move towards greater secularism and self-expression, whereas economic decline (especially in ex-Communist countries) precipitated a greater emphasis on traditional and survival values. These trends are consistent with Inglehart’s (1990) model of materialism-to-postmaterialism two-dimensional model of cultural change, which suggests that economic prosperity brings about a shift from survival to quality-of-life concerns. More recently, Inglehart et al. (2008) extended Inglehart’s (1990) model to suggest that economic prosperity, together with democratization of society and social liberalization, increases the availability of various social opportunities to make a free choice, which in turn enhance sense of freedom. They argue that it is this sense of freedom that is closely linked to subjective well-being.

Although longitudinal research typically uses survey methods, this is not the only research design available. Greenfield et al. (2003) conducted a unique longitudinal study of a Zinacantec Maya community in Chiapas, Mexico. After studying weaving apprenticeship and representational style of weaving patterns in 1969 and 1970, Greenfield and her colleagues went back to the same site and examined the same variables in 1991 and 1993. The 20 year time span saw a greater penetration of commercialization and market economy into the area, and enabled the researchers to observe changes in cognition and practice. They postulated that there are two cultural models of teaching/learning. In a conservative model of apprenticeship, the master guides an apprentice’s learning, the apprentice does not have an opportunity to make a mistake and learn from it, but rather the apprentice acquires the master’s skills with little change and variation. In contrast, a progressive model encourages the learner to acquire skills by trial-and-error, by making mistakes and correcting them. This provides a greater opportunity for the learner to be more independent and innovative. The former is more adaptive when errors are costly, but the latter is possible when errors are less costly. They argued that the conservative model is more adaptive in the subsistence economy, whereas monetary economy and formal schooling tend to promote the more trial-and-error learning. As well, the traditional subsistence-based community would encourage “concrete exchange of goods” whereas market-based economy would encourage “abstract exchange of symbolic equivalents,” and as a result, the former resulting in more concrete representations whereas the latter, more abstract representations. Their empirical findings suggested that not only was there a historical change in more independent, trial-and-error learning and abstract representational style, but also it was mediated by the family’s engagement with commercial and market economy. As well, more independent learning style correlated with more abstract representational style.

***Potential uses of cross-cultural comparative method for cultural dynamics***. Although the cross-temporal methods have been highlighted so far, cross-cultural comparative methods can be fruitfully used to shed light on cultural dynamics as well. Most theorists of culture and psychology recognize the ecological and socio-political environment as potential causes of cultural dynamics. That is, the culture of a group may form, persist, and change because it is adaptive to do so given the natural and human-made environments in which the group is situated (e.g., Cavalli-Sforza and Feldman, 1981; Boyd and Richerson, 1985; Berry et al., 1992; Triandis, 1994; Cole, 1996; Kagitcibasi, 1996; see Oishi and Graham, 2010, on a recent synthesis). The human-made environment is that part of the environment that is structured by human activities, which of course are informed by culture as defined here. Parenthetically, some theorists (e.g., Herskovits, 1955; Triandis, 1994) call this culture, but it is a definitional matter that depends on theorists’ choice and perspective. A more recent development in evolutionary biology (niche construction model; e.g., Laland et al., 2000; Odling-Smee et al., 2003) too suggests that culture helps construct the human-made environment, which in turn affects the natural environment. Nonetheless, these environments in turn affect culture. Culture (as well as genetic composition) is an adaptation to both the natural and human-made environments; as the environments change, so should culture.

From this perspective, then, it is possible to infer some elements in the natural and human-made environments as causes of the formation and change of culture by using cross-cultural comparative methods. For instance, Nunn (2008) used historical records to estimate the number of slaves exported from African countries between 1400 and 1900 (an element of the human-made environment), and correlated it to per capita GDP in 2000 (a measure of human activity). There was a robust negative relationship, suggesting that enslavement of the populace in those regions by internal warfare, raiding, and kidnapping (often across ethnic boundaries, but sometimes even within them), collapse of the traditional social institutions, and political instability engendered mistrust (Nunn and Wantchekon, 2011) and made it difficult to establish productive economic activities. Becker and Woessmann (2009) too used comparative data to argue that the Protestant regions of Prussia showed greater economic progress that the Catholic regions not necessarily due to religious beliefs *per se*, but by the literacy promoted by the Protestants. Perhaps most closely relevant to culture and psychology, Gelfand et al. (2011) reported that cultural tightness – the extent to which cultural norms are enforced and their violations punished – is correlated with population density of a country in 1500, suggesting the need to cooperate and coordinate economic activities earlier in history was a potential cause of tightness. The utility of cross-cultural comparative methods notwithstanding, it is still important to take a cross-temporal perspective seriously in cultural dynamics as cross-sectional data may produce a spurious correlation (e.g., Becker and Woessmann, 2013).

***Findings from cross-temporal methods***. Although the cross-temporal meta-analyses and cross-temporal analyses of running surveys from the US data appear to suggest a general trend towards a greater individualism, there is evidence for some countervailing trends as well. When research is extended to Japan, there is evidence for increasing trends for both individualism and collectivism. In addition, when broader cross-national historical trends are examined, it is difficult to draw a strong conclusion about the historical trends in a clear cut way. In many ways, the historical trends in attitudes and values are embedded in the historical transformation of human society and culture, which may be called *globalization* – greater exchange of information and people, greater integration of financial and economic activities, and greater interdependence among peoples in political, societal, and environmental domains. More nuanced understandings about cultural changes may be afforded by greater understandings about the *mechanisms* of cultural dynamics, that is, what processes maintain or transform the current distribution of cultural information in a human group.

#### Cross-generational method

A central mechanism of cultural dynamics is cultural transmission, that is, how cultural information is transmitted from one person to another (Kashima, 2008). Cavalli-Sforza and Feldman (1981) distinguished three types: *vertical* transmission from parent to child, *horizontal* transmission from peers, and *oblique* transmission from an older generation to a younger generation with no genetic link. The first one is cross-generational method, in which a person’s cultural characteristic is examined and correlated with the characteristic of his or her parents or others from an older generation that he or she is affiliated with. Cavalli-Sforza et al. (1982) provide one of the earliest examples of this research design. They distributed a survey asking undergraduate students about a variety of cultural traits including their religion, politics, values, attitudes, beliefs, and habits; they also examined these students’ parents as well as same sex and opposite sex friends. They found that the rate of vertical transmission varied greatly from one domain to next – religion and politics (e.g., Democrat, Republican) were more strongly transmitted than beliefs (e.g., reading horoscope) and habits (e.g., drinking coffee or tea), for instance. Interestingly, they found that paternal and maternal transmissions were mostly additive – having both parents possessing the same cultural characteristic did not add any extra strength to vertical cultural transmission.

There have been more recent investigations of vertical cultural transmission using a similar method. Although there is a great deal of nuances in findings and theory (see Schönpflug, 2009), two major insights for cultural dynamics have emerged. First, cultural information differs in ease with which it is vertically transmitted. In particular, *social function* of cultural information may be a significant factor. In particular, drawing on Campbell’s (1975) theory of genetic and cultural evolution of morality, *ceteris paribus,* Phalet and Schönpflug (2001) and Schönpflug (2001) argued that cultural information that promotes *social integration* (e.g., collectivist values such as traditionalism, security, and conformism; see Schwartz, 1994) tend to be transmitted better than the values that emphasize *individual autonomy* (e.g., individualist values such as self-direction, stimulating life, hedonism). Consistent with this, when Schönpflug (2001) examined value transmission from father to son among Turkish immigrants in Berlin and a Southern German city of Lake Constance, as well as Turks living in Istanbul, Turkey, she found that collectivist values were likely to be vertically transmitted, but not many individualist values were not. Similarly, in their examination of Turkish immigrants in Germany and Turkish and Moroccan families in the Netherlands, Phalet and Schönpflug (2001) reported that they too showed a tendency to vertically transmit social integrative attitudes to parent–child relationship. Sabatier and Lannegrand-Willems’s (2005) three-generation cultural transmission research too found that collectivistic values are transmitted among French women, not only from grandmother to mother but also to granddaughter as well.

Nonetheless, it is important to consider the factor of environmental stability as a moderator of this general tendency. Kameda and Nakanishi (2002) showed that, consistent with Boyd and Richerson (1985), there is an overall adaptive advantage to social learning (i.e., learning from others by imitation or mimicry) when the environment with which people interact is stable; however, when the environment changes (and therefore action-reward contingencies change), the advantage of social learning declines, and individual trial-and-error learning becomes relatively more adaptive. Likewise, when a group’s environment is rapidly changing, collectivist values may be transmitted less, and individualist values may be transmitted more. Consistent with this line of reasoning, Boehnke (2001) reported that, in the midst of a transition from communist past to capitalist market economy, East German families showed a sign of transmitting individualist values such as hedonism and stimulation, but not so much for collectivist values such as conformity and security.

Second, the generation that receives cultural information plays a critical role in vertical cultural transmission. Grusec and Goodnow’s (1994) two-step model illustrates this point most clearly. According to their model, cultural transmission is a function of a child’s accurate perception of a parent’s beliefs, attitudes, and values, and the child’s acceptance or rejection of the perceived parental stance. Unless children accurately perceive what their parents value and accept it as their own, they would not adopt the same value. Zentner and Renaud’s (2007) findings provide support. Children’s accurate perception and acceptance of their parents’ ideal self was both necessary for vertical transmission of ideal self (the life goals and personality characteristics one regards as ideal for oneself). In particular, they showed that the congruence between parents’ ideal self and children’s ideal self was predicted by an interaction between the accuracy of children’s perception and acceptance of their parents’ ideal self. It is noteworthy that they also showed that the parents’ wish to transmit – what they regard as ideal for their children – also played a significant role, and nurturing parenting practices tended to facilitate, but restricting parenting practices, inhibit, vertical transmission by affecting each of the steps of the vertical transmission processes. Knafo and Schwartz (2009) also reported empirical evidence in line with Grusec and Goodnow’s two-step model.

In the end, vertical cultural transmission is present for some attitudes and values (for instance, Zentner and Renaud, 2007, estimated this to be about 0.10 for life goals and 0.20 for personality traits in their data), but not overwhelming. It suggests a significant role that other cultural transmission processes – oblique and horizontal – play in cultural dynamics. Boehnke (2001; also see Boehnke et al., 2007) explored this by examining the effect of what he called Zeitgeist on a younger generation’s value preferences. In a two generation data set where children’s and their parents’ values were measured, he randomly selected for each child a father, a mother, and another child who are unrelated, and examined the correlation between the aggregated value score of these three unrelated people and the child’s value. The size of Zeitgeist effect was as large as a mother’s and even larger than a father’s effect. In addition, the Zeitgeist effect was greater when parents’ values diverged, suggesting that oblique and horizontal transmissions are greater when vertical transmissions are weaker. Vedder et al. (2009) extended this work and examined the effects of oblique and horizontal transmissions of family obligation values in young immigrants and nationals in eight European countries as well as the USA and Australia. They found that vertical transmissions were strong for both immigrant and national youths; for immigrant youths, horizontal, and oblique transmissions from their own ethnic group were both significant, whereas for nationals, only oblique transmissions were significant. However, there was no evidence of cross-ethnic oblique or horizontal transmissions. Neither immigrant nor national youths were influenced by their outgroup others of their parents’ or their own generation.

#### Experimental simulation method

The Zeitgeist method is a creative solution for the problem of examining oblique and horizontal cultural transmission. However, it provides somewhat indirect evidence. Although it shows that people of a given generation show similar attitudes and values, and therefore there is a cohort effect, it does not enable a direct examination of the micro-level process by which a person transmits, and another person receives, cultural information. Experimental simulations of cultural transmission are probably the most suitable research design for this purpose.

There have been at least two broad classes of experimental designs. One is the *method of serial reproduction* (Bartlett, 1932; Allport and Postman, 1947; see Kashima, 2000b, for historical background). Cultural information in a variety of forms (e.g., written text, oral communication, picture, artifact) is used as a stimulus in the design. One person receives it, who then transmits it from memory to a second person, who in turn transmits it to a third person, and so on (see Kashima and Yeung, 2010). The method was used in a variety of context in the past, but more recently revived in experimental examination of stereotype maintenance (e.g., Bangarter, 2000; Kashima, 2000c; Thompson et al., 2000) and value maintenance (Imada and Yussen, 2012). For instance, Kashima (2000c) constructed a story that contained information that is consistent and inconsistent with gender stereotypes. He showed that, although people reproduced gender stereotype inconsistent information immediately in the first and second generations of reproduction, stereotype consistent information was more likely to be passed on in communication chains, eventually becoming a more prominent part of the story.

This finding largely replicates what Bartlett (1932) called conventionalization – information passed on through serial reproduction chains is eventually transformed to be similar to what is shared within the group from which the members of the reproduction chains were taken. Kurz and Lyons’s (2009) findings corroborate this overall observation. They found that this happens most prominently when outgroup stereotype relevant information is communicated among ingroup member. Subsequent research using this method has shown that the tendency to transmit stereotype consistent information is likely driven by the transmitters’ perception that most members of their ingroup endorse the cultural stereotype (Lyons and Kashima, 2003) and that this relates to the social function of stereotype consistent information, that is, communicating information that is consistent with the shared and commonly endorsed cultural information maintains or even strengthens the social connection between the transmitter and the receiver of the information (Clark and Kashima, 2007). Taken together, it shows that more socially integrative information is more likely transmitted and thus likely to contribute to cultural maintenance. More recently, however, Lee et al. (2014) used the method of serial reproduction to illustrate a mechanism of cultural formation – when initially unbiased information about intergroup conflicts was passed through serial reproduction chains of members of one of the groups involved in the conflicts, the story became increasingly more biased in favor of their own group. Similarly, Martin et al. (2014) also reported that initially randomly information became more structured and stereotype-like when it was serially reproduced.

A second experimental paradigm was used by Jacobs and Campbell (1961), which may be called the *method of joint participation*. They showed that people’s joint participation in a group task can act as a medium of cultural transmission. In one of the conditions, a group of three people participated in Sherif’s (1935) auto-kinetic effect paradigm; however, there was only one naïve participant and the other two were confederates, who publicly reported large autokinetic movements; and the naïve participant eventually reported a similarly large autokinesis. One of the confederates was then replaced by a naïve participant, then the second confederate was replaced, and so on, with each generation bringing in a fresh naïve participant. They reported that the cultural practice to report an unusually large autokinetic movement remained even after all the members of the initial group were replaced by naïve participants. This paradigm showed that not only is the co-presence of group members a powerful influence on cultural transmission, but also joint participation in a task was sufficient for the effect to occur.

This paradigm was used by Zucker (1977) to show that cultural transmission can be facilitated by institutionalization of the group task. In one of the conditions, participants were told to regard the autokinetic experiment as something akin to an organization or an office, in which there are certain personnel, different tasks are distributed among different people, and even if one person retires and a new person enters, and so its members may change constantly, the organization and the office remains the same, thereby emphasizing a temporal continuity of the operation. Zucker found that these conditions strengthened the cultural transmission process when compared to the condition that replicated Jacobs and Campbell’s (1961) original study.

More recently, Kashima et al. (in press) used the method of joint participation to show that implicit attitudes can be transmitted as well. In their experiment, participants were told to imagine that they were on an alien planet, and to learn its inhabitants’ culture. One participant was trained to perform a simulated foraging task in which he or she is to collect certain alien fruits and discard others; consistent with their earlier study (Laham et al., 2013), he or she acquired corresponding implicit attitudes – positive attitudes for those fruits that they collected and negative attitudes for those that they discarded. The trained participant was paired with another untrained participant, so that they can participate in a joint foraging task. The trained participant continued to forage as before. However, the untrained participant was given the instruction to collect or discard only a subset of the fruits that they encountered; this participant had to decide whether to collect or discard those fruits for which they received no instructions. The untrained participant spontaneously learned to collect or discard by imitating the trained participant’s action. In addition, he or she also acquired the implicit attitudes that were congruent with the trained participant’s attitudes. Further analyses suggested that this transmission of implicit attitudes occurred through the untrained participant’s observation of the trained participant’s action – they observed the trained participants collecting and discarding fruits, inferred that the latters’ attitudes, and turned these attitudes their own. Just like cross-generational studies showed that cultural transmission occurred through children’s observations of their parents’ values and acceptance of them, this experimental simulation too suggested that the cultural learner’s cognitive processes plays a critical role in cultural transmission.

#### Formal modeling and computer simulation method

The designs examined so far are all empirical in that they all make use of observations of human action. However, non-empirical means have been used to investigate micro-macro linkages in cultural dynamics. For example, both Cavalli-Sforza and Feldman (1981) and Boyd and Richerson (1985) used mathematical equations – primarily difference and differential equations – to describe micro-level cultural transmission processes, and their long-term and macro-level consequences are explored by solving for an equilibrium. That is, when the expected temporal change in culture approaches zero, the cultural system described by the equations becomes stable, and the overall prevalence of cultural characteristics within a population becomes self-perpetuating. By investigating various conditions in which equilibria are reached, the researchers could examine how cultural dynamics unfold over time. More recently, however, models of cultural processes have become more complex, and it has become difficult to examine the macro-level implications of micro-level cultural processes by analytically solving equations. To go around this problem, computer simulation models have been used for the purpose, and they are often called agent-based models. McElreath and Boyd (2007) give an excellent treatment of mathematical models of cultural evolution. Railsback and Grimm (2012) provide a practical guide for agent-based modeling approaches. There are two prominent lines of work: evolution of cooperation and cultural diffusion.

***Evolution of cooperation***. One of the enduring puzzles for Darwinian theory is the evolution of cooperation. Although cooperation with another individual often incurs cost, and non-cooperation (often called defection) brings more benefit to the individual than cooperation. A well-known example is a prisoner’s dilemma. Two robbers are caught by police, interrogated separately, and offered a choice between (c) not confessing and keeping silence, and (d) confessing to the crime and getting a lighter sentence. If both choose (c) and keep silence (i.e., cooperate), they go free; if one chooses (c) and the other chooses (d) (i.e., defect), then the defector gets a lighter sentence, but the co-operator gets a longer sentence; and if both choose (d), then both get a longest sentence. The sentence lengths are rigged, so that whatever the partner chooses (i.e., cooperates or defects), a robber is always better off defecting. Under this type of situation, how can cooperation as a cultural practice become prevalent within a human group?

A theoretical framework called evolutionary game theory (Maynard Smith, 1982) has been used to investigate this question. Roughly speaking, the framework assumes that all agents have two or more strategies at their disposal, one of which is cooperative and others are less so. A game is pre-defined so that the outcome for each agent is determined as a function of the combinations of which strategy each agent selects. For instance, in the case of a prisoner’s dilemma game, what sentence one gets depends on whether one cooperates or defects and whether the partner cooperates or defects. This strategy-outcome contingency determines the incentive structure of the game. Again, in the case of prisoner’s dilemma, defection is always better off than cooperation, and so defection is a dominant strategy. What this means is that defection is an evolutionarily stable strategy – all agents will become defectors and no one will cooperate. However, there are a number of circumstances that enable cooperation to become evolutionarily stable (Nowak, 2006a). This article cannot do justice to this extensive and complex literature. Readers are referred to Nowak (2006b), who provides a relatively recent brief survey.

***Diffusion of culture***. The notion that cultural information diffuses has a long history (Kashima et al., 2008). Anthropologists in the early 20th century discussed the distribution of culture around the world in terms of cultural diffusion (Smith, 1946); the method of serial reproduction was invented to simulate the process of cultural diffusion (Kashima, 2000b); and technological innovations as cultural artifacts have been conceptualized as diffusion processes (Rogers, 2003). Taga and Isii (1959) may be one of the earliest attempts to formally model a diffusion process. Since then, some have developed into more sophisticated formal models especially in the context of market penetration by new products (e.g., Niu, 2006).

However, it was Nowak et al. (1990) that marked the recent upsurge of interest in simulations of cultural diffusion processes. They modeled each agent as having one of two possible states, which can be interpreted as pro- or anti-stances on an issue, positive or negative attitudes, or belief or disbelief. Each agent occupies one square in a lattice, and maintains or changes its state as a nonlinear function of the supportive or opposing communications it receives from its neighbors. When the agents are initialized to have random states, and its algorithm is used to update each agent’s state, the agents eventually settled in a steady state where no agents changed their states. Under some cases, the agents clustered together to form a group with the same opinion state. This was interpreted as a formation of a culture (Latané, 1996; see Harton and Bullock, 2007, for a review). The dynamic social impact model provided an initial demonstration that agent-based models are a useful method to examine the implication of micro-level cultural processes for macro-level cultural dynamics. In particular, it was known that when social influences were understood to be linear, rather than nonlinear, the type of opinion clustering that exists in society could not be produced. This demonstration showed that micro-level cultural processes should be nonlinear. Cullum and Harton’s (2007) empirical study of the evolution of college dorm cultures largely supported the theoretical predictions.

Despite the importance of this line of work, it became clear that modeling cultural information as a single binary attribute was somewhat limiting. Axelrod (1997) proposed a multi-attribute model of cultural information that has become a pre-eminent platform for cultural diffusion modeling. In his model, cultural information is represented as a vector with more than two (and often more than five) elements, each of which can take one of more than two values (and again often more than five values). At any given point in time, each agent has a certain pattern of cultural information as specified by this vector. The process of cultural diffusion is also somewhat more complex – when two agents interact, one element in their culture vectors is changed, so that they become culturally more similar. This change models cultural transmission – one agent’s cultural element is transmitted to the other agent, and as a result, the latter’s culture vector changes. The likelihood of transmission, however, depends on how similar their cultural patterns are to begin with. The more similar they are, the more likely they exchange cultural information. Nothing in the model promotes divergence of the interacting agents’ culture vectors, and so they tend to form clusters of agents with the same culture, and often the whole population of agents end up having the same culture. However, more often than not, the agents form different cultural clusters, preserving cultural diversity.

There have been an explosion of research based on the Axelrod model (A search on June 12, 2014 returned 422 citations on the Web of Science), and the citing papers are found not only in social sciences, but also in evolutionary biology, computer science, physics, and the like (see Castellano et al., 2009, for a review). For example, some research examined cultural dynamics and long term formation of cultural diversity by exploring the effects of mass media (e.g., González-Avella et al., 2005; Rodríguez and Moreno, 2010), different models of cultural transmission processes (e.g., Kuperman, 2006; Flache and Macy, 2011), and implications of static (e.g., Klemm et al., 2003; Xiao et al., 2009; Guerra et al., 2010) as well as dynamically changing social networks and agent movements within space (e.g., Centola et al., 2007; Gracia-Lázaro et al., 2009). Some have considered the implications of the model for the possibility of maintaining cultural diversity in the face of globalization (e.g., Greig, 2002; Pfau et al., 2012).

### SIMILARITIES AND DIFFERENCES AMONG RESEARCH DESIGNS

The four broad classes of research designs discussed so far differ in at least three important respects: time scope, level of analysis, and direction of inference. **Table 1** presents a rough summary of similarities and differences. Cross-temporal methods are typically used to examine long- to medium-term, macro-level trends and trajectories of cultural dynamics. Some have documented cultural changes over centuries, and others, decades. Cross-generational methods are used to examine cross-generational transmissions of cultural information medium-term – from one generation to next, or at most three generations. Experimental simulations are typically for investigations of the micro-level mechanisms of cultural transmission in a short-term although some have attempted to generalize their findings to longer-term processes, namely, cultural transmissions across generations (e.g., Caldwell and Millen, 2009). These three are all empirical research designs in that they are for collecting empirical observations and testing theoretical propositions. In contrast, formal models and computer simulations are not for data collection, but for generating theoretical propositions. Starting with a set of assumptions and propositions about the mechanisms of cultural dynamics, their macro-level, typically long-term, and global implications (e.g., prevalence of cooperation, cultural diversity) are examined. In this sense, they are deduction machines that enable researchers to explore implications of their theoretical assumptions and propositions, but they cannot be used to test their theory.

**Table 1:** Research designs.

	Time scope	Level of analysis	Inference
Cross-temporal method	Long	Macro	Empirical
Cross-generational method	Medium		Empirical
Experimental simulation	Short	Micro	Empirical
Formal model and computer simulation		Micro–macro Linkage	Deductive

*There are some blank cells, representing that there is no clear implication.*

#### Complementarity of research methods

These research designs complement each other. Formal models and computer simulations can be used to generate new hypotheses, but the other empirical designs are needed to test them. The models typically make assumptions about the mechanisms of cultural transmission (e.g., imitation), but whether these assumptions are correct can be tested by experimental simulations of cultural dynamics. For instance, Kashima et al.’s (in press) findings suggest imitations may be a mechanism for cultural transmission of behavioral practices, but inferences appear to be critical for cultural transmission of attitudes. However, long-term and macro-level implications of micro-level dynamics are difficult to see without formal models or computer simulations. Experimental investigations have suggested that cultural information consistent with widely shared cultural information is more likely to be transmitted (e.g., Clark and Kashima, 2007); however, it takes a computer simulation model like Axelrod’s (1997) to see that this process, which tends to enhance cultural convergence, can nonetheless generate global cultural diversity under some circumstances – even when globalizations are underway (e.g., Pfau et al., 2012).

Nevertheless, without cross-temporal investigations, it is impossible to conclude whether cultural diversity is being maintained or cultural convergence is occurring. Cross-temporal investigations appear to show both the persistence of cultural traditions and the instigation of new cultural orientations (e.g., Inglehart and Baker, 2000). Furthermore, it takes cross-generational investigations to find what cultural elements are in fact transmitted from the current generation to the next under what circumstances (e.g., Boehnke, 2001; Phalet and Schönpflug, 2001), whether or not oblique and horizontal cultural transmissions are occurring within and across cultural borders (e.g., Vedder et al., 2009), and so on.

#### Issues related to methods of observation

All the empirical research designs involve observations of human action or its outcomes (e.g., cultural artifact such as arrowhead produced by a series of human action). In cross-generational and experimental simulation research, researchers (or their trained observers) are directly engaged in the observation. However, for cross-temporal research, because of its long-term time scope, researchers are not always the observers of the target phenomenon. Instead, as explicated by Runkel and McGrath (1972), the intervening step of record taking and record keeping is critically involved.

**FIGURE 1:**
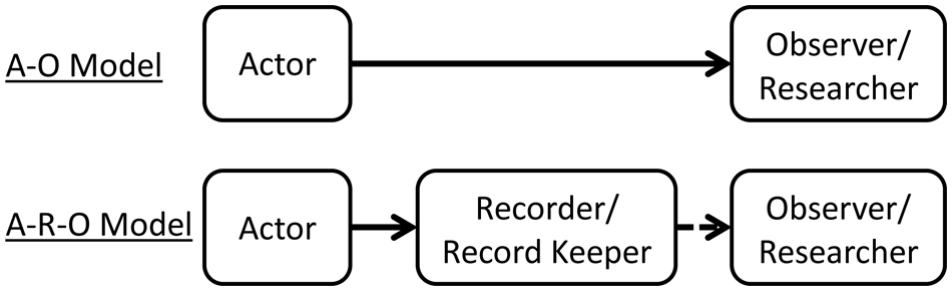
**Models of observation method**.

**Figure 1** schematically represents this issue. In typical social science investigations, the A–O model suffices; however, an explicit recognition of the role of recorder (A–R–O model) is necessary particularly in cross-temporal methods. For example, in cross-temporal meta-analysis, when Twenge (1997) examined the cultural dynamics of attitudes towards women, she was not observing the participants’ responses in her research. She was using the results reported by some other researchers, who acted as recorders and record keepers of the original participants’ responses. In cross-temporal archival research, when Oishi et al. (2013) examined US presidents’ speeches, he was using the records kept by the US bureaucracy. The distinction between the A–O and A–R–O models may seem akin to the contrast between primary and secondary sources in historical research, but they are different. The recorder/record keeper is usually regarded as the author of a primary source. For instance, most historians would regard the published research used by Twenge (1997) as primary sources, but the authors of the published research is regarded as a recorder in the A–R–O model.

Most of the familiar discussions about validity and reliability apply to cultural dynamical research methods; however, the intervening process enacted by the recorder/record keeper introduces a number of methodological issues that are perhaps unique to cultural dynamics research. First, the *representativeness of the records* used to obtain data needs a careful examination. It is about the A–R process in the A–R–O model. For instance, the Google Books corpus is not representative of all the books published in a given language because the collection of the books is largely dictated by the acquisition policies of the libraries participating in the project. More generally, if there is any bias in the entry, survival, and retrieval of records relative to the totality of cultural information available in the group at a given point in time, it can be a threat to the construct validity and bias the conclusions drawn from the research. Webb et al. (1981) lists a number of examples: Roman tombstones as a measure of life expectancy, Congressional Records, *Who’s Who*, deaths reported in obituaries, records of office holders, etc. These are records that were or have been kept by recorders for the purposes other than the research. They are typically records of economic transactions, political decisions and statements, and other forms of institutional activities. The recorders keep this type of information for their own purposes, and for these reasons, there can be some biases: selective deposit and selective survival.

Perhaps the most acute example comes from archeological studies. For instance, O’Brien et al. (2001) examined Palaeoindian artifacts, in particular, projectile-points (e.g., spear, arrow), which were left behind by the Amerindians living in the southeastern United States around 11,500–10,000 BP^10^. Various projectile-points found, cataloged, and kept can then be dated, measured, and classified in terms of their physical characteristics along measurable dimensions (e.g., location of maximum blade width, base shape, length/width ratio), thus constructing a design space. These data can then be used to conduct what is known as cladistics, a study of common ancestry and lineages in biological evolution. Likewise, archeologists can describe which type of projectile-point gave rise to which type, tracing the direction and pace of technological change with regard to projectile-point within the design space. It is contended that this type of analysis allows researchers to measure and explain cultural changes (Lyman and O’Brien, 2000). However, if some cultural artifacts are lost for some reason, this type of research can yield seriously biased conclusions.

Second, another critical issue is the *representativeness of measures*, namely, words, terms, and phrases used to measure a given construct. It is about the R–O process in the A–R–O model. For instance, Researchers typically use the vocabulary of contemporary words and phrases as a starting point, and use a contemporary sample’s or researchers’ own intuitions to select the search terms, for instance, for the Google Ngram Viewer. However, these procedures do not guarantee the representativeness. More generally, given a certain construct of interest, the meaning space for that construct needs to be sampled to ensure that the selected terms etc. appropriately reflect the construct of interest. Again, a bias in this sampling procedure (i.e., cherry picking search terms) can threaten the construct validity of the measurement.

The next set of methodological issues stems from the cross-temporal nature of cultural dynamical research, that is, *historical relevance*. This comes in two versions. One is *historical relevance of measures*. A measure of a theoretical construct of interest may be valid and reliable at one point in time, but may cease to be so at another. An excellent example comes from a well-known Princeton trilogy of US ethnic stereotypes (Katz and Braly, 1933; Gilbert, 1951; Karlins et al., 1969) in which Princeton undergraduates’ stereotypes about various ethnic groups including Black stereotypes were examined in the United States. The constructs of interest were ethnic stereotypes, but their measures had to change over time. In measuring Black stereotypes, the terms included in the 1933 study (e.g., physically dirty, naïve, slovenly) were dropped in the 1951 survey presumably due to their general irrelevance. Although the 1969 study brought those terms back in, only less than 5% of the participants selected them as stereotypes anyway, suggesting that they were no longer relevant after three decades. Instead, the 1951 study included “pleasure loving” as another term, which was selected by 19% of the participants then. The proportion of its endorsement as stereotypical increased to 26% in 1969 (see Devine and Elliot, 1995).

The other issue is *historical relevance of constructs*. The meaning and importance given to certain theoretical constructs could change over time. In retrospective cross-temporal research, theoretical constructs of contemporary relevance may not have had any relevance in the past. Or the meaning of a construct, even though the same term or word is used, may have been very different in the past. In prospective cross-temporal research, constructs of interest at one point in time may become less important later; conversely, constructs unimportant (or even non-existent) now may gain importance in the future.

In the case of prospective cross-temporal research, the only solution seems to be dynamically changing measures or constructs, dropping irrelevant ones and including relevant ones. Nonetheless, an obvious problem is lack of comparability over time. Comparable data may be available only from one portion of the time series. At times, the absence of data (by inclusion or exclusion of constructs or measures) may provide useful information. For instance, the absence of data may be interpretable as indicating historical changes in relevance (e.g., “physically dirty” may have been relevant in the 1930s, but irrelevant in the 1950s, presumably due to a general increase in hygiene, health standard, and socio-economic conditions of the US population). However, the absence of data cannot guarantee that they are equally irrelevant over time. When a construct is changed or an operationalization is modified, researchers must have made the decision by some criteria (e.g., pilot study, intuition), but these criteria are not guaranteed to be equivalent.

#### Model of action and interpretation

The last set of issues – historical relevance of measures and constructs – touches on a pernicious epistemological issue in philosophy of social science. Cultural psychologists are typically concerned about cultural information that is *meaningful* to the actors who produce the phenomenon of research interest. To use a short hand, most cultural dynamics research examines *action* understood as meaningful behavior. However, meaning has often been missing from psychological research, and how to handle it has been a contentious epistemological issue (see Kashima, 2000a).

To spell out this issue in the context of cultural dynamics research, an explicit model of action and interpretation seems desirable. The following is an attempt to sketch it out based on the existing literature (e.g., Ricoeur, 1971; Taylor, 1971; Shweder, 1990; see Kashima, 2005, for a review and discussion). To begin, let us assume the following.

1. Action is typically motivated and interpreted in terms of the actor’s meaning of the action, which is intelligible against the actor’s understandings about the social and non-social reality.
2. Action is performed against the background of social and non-social reality.
3. Social reality is culturally constituted.

An action as a meaningful behavior can be interpreted by the actor or the audience of the action in terms of a meaning (or cultural information) in a culture. Meaning here is defined in relation to the whole of culture (i.e., a set of cultural information available to a group as defined earlier; Taylor called it “field”). The audience interprets the actor’s action in terms of this meaning within these “webs of significance (Geertz, 1973, p. 5).” It is important to note that the actor can be the audience as well. Consistent with this notion, recent research on action execution and comprehension suggests that the same underlying neural substrate appears to be involved in both one’s own planning of a voluntary action and an understanding of someone else’s motor action, and the actor’s experience of intending to perform the motor behavior and the audience’s interpretation of it appears to be closely associated with this neural substrate (e.g., Rizzolatti and Craighero, 2004) although evidence for a definitive conclusion still eludes the research (Kilner, 2011).

**FIGURE 2:**
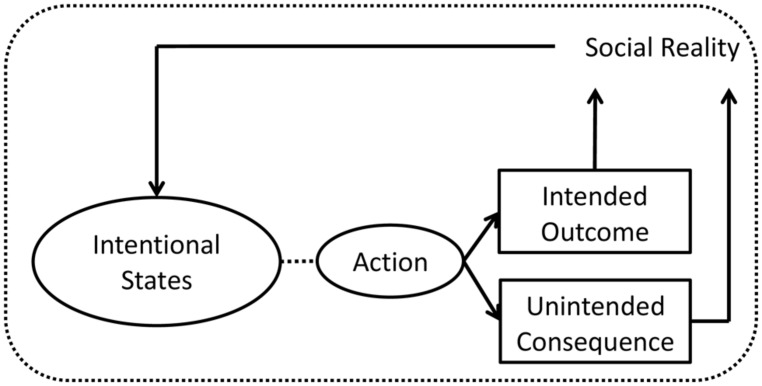
**Model of action**.

Nevertheless, the actor’s action occurs within the context of social and non-social reality. Because the meaning of an action is typically understood in terms of intentional states such as beliefs, desires, and intentions, as well as emotions, it is usually safe to assume that the reasons for action (beliefs and desires) and the triggers of emotions derive from the social and non-social reality as the actor understands it (e.g., Ross and Nisbett’s, 1991, principle of construal). In turn, the actor’s action shapes social reality via its intended outcomes and unintended consequences (see **Figure 2**).

In this sense, the meaning of an action for the actor and the audience is embedded in the world (indicated by shorthand “social reality” in **Figure 2**) imbued with cultural meaning. Taylor (1971) suggests that the meaning of action cannot be defined without reference to the terms for intentional states such as emotions, beliefs, desires, and intentions, which are all in turn defined in reference to other intentional states. And hence, it is a self-referencing, self-contained, and closed meaning space. This is what Shweder (1990) called the *intentional world*. In cross-cultural research, researchers can often consult with colleagues or informants from a cultural group that they are studying. However, in cross-temporal research, there may not be any colleague or informant available. They may be long gone. Researchers may at times have lived through some of the historical period that they are investigating (e.g., Twenge, 2011), but that is not always the case. Whereas backtranslation with decentering may be a standard procedure for checking cross-cultural equivalence, there may be no one who can backtranslate researchers’ constructs or measures in cross-temporal research.

How is it possible to understand meaningful action in the past without an informant? This is the crux of the problem. Taylor (1971) suggests that interpreters (i.e., researchers) with a real experience of living in the cultural group’s intentional world can understand the meaning; however, if live experiences are necessary for understanding meaningful action, cross-temporal research with a long time scope becomes impossible. In contrast, Ricoeur (1971) argues that this intentional world has external referents, and is anchored in the physical reality of a sort. This is because the intentional world is constituted by what Searle (1969) called *constitutive rules*, which stipulate a symbolic function of physical stimulus, X (physical stimulus) counts as Y (meaning) in context C.

Whether constitutive rules are explicitly understood and used by all actors is debatable (Kashima, 2004), but humans learn to perform actions against the social reality that *presupposes* the constitutive rules. To clarify this point, let us imagine a future archeologist who is investigating the cultural dynamics of the 21st century Earth. So, even if a 5-year old who gives a US$10 bill to a shopkeeper to purchase a chocolate bar doesn’t know the constitutive rule – that type of green piece of paper (X) counts as money (Y; US$10) in the 21st century United States of America (C) – his action can be interpreted in terms of this constitutive rule. To the extent that this constitutive rule is recoverable from elsewhere (e.g., US legislation, other documents, etc.), the teenager’s action to hand over the green piece of paper to the shopkeeper, and to receive a chocolate bar (and some changes) in return becomes intelligible in the intentional world.

These symbolically expressible constitutive rules, and the fact that human actions are performed by human bodies in time and space (embodied in this sense as in Taylor, 1971) and the recent neuroscience insights that the human brain-body appears to represent one’s own and others’ actions in the same neural substrates (this is something that Taylor, 1971, did not know) give us sufficient chances for researchers of the present to interpret the actors’ cultural world of the past even if they are separated in time and space. This model suggests that what is required is the practice known as “hermeneutic cycle,” that is, to arrive at an interpretation of action meaning by cycling from the object of inquiry to other contemporary texts and contexts and back again to the object of inquiry, only to restart the same process again and again until some validity criterion is reached. By careful explication of constitutive rules and their methodical application, the model suggests that it is possible to arrive at an increasingly valid interpretation of a meaningful action.

## CONCLUDING REMARKS AND FUTURE DIRECTIONS

Methodology for research on cultural dynamics is diverse. Cross-temporal methods using archival data, past research, running surveys, and longitudinal surveys can bring out macro-level cultural change in long- to medium-term. Cross-generational and experimental simulation methods can shed light on medium- to short-term mechanisms of cultural dynamics. Formal models and computer simulations can help consider complex implications of models and mechanisms, and link micro-level mechanisms with macro-level dynamics. By combing these complementary methods, cultural dynamics research should be able to bring out new insights about cultural change and human experience.

The empirical methods of cultural dynamics have much in common with any other social science research. A number of validity and reliability concerns need to be addressed in order to support or challenge a theoretical proposition or statement, which the researcher wishes to test and which typically involves some form of generalization about a social phenomenon of interest. There are well known issues of construct validity, internal and external validity, and the like as well as threats to them (e.g., Campbell and Stanley, 1963; Cook and Campbell, 1979; Brewer, 2000; see Reis and Judd, 2000).

Nonetheless, it brings into focus two classes of validity issues that are rarely problematic in typical psychological research. First, because it deals with meaning, the question about interpretation and how it differs from causal explanation – an enduring epistemological issue that cultural psychology brought to the forefront – is also present. In addition, because of its cross-temporal nature, the relation between researchers and the object of inquiry is often more complex. The existence of the recorder and record keeper should be explicitly recognized and its potential role as an enabler and a bias needs to be considered.

The most pernicious of all the methodological issues stems from the interaction of the two – the fact that cultural dynamics is about meaning over time. Is the measurement procedure valid across time? Even if a measure may be valid now, there is no guarantee it was so in the past or will be in the future. Is a construct relevant across time? A construct meaningful at one historical time may not be meaningful at another period of time. Furthermore, this issue can potentially bring up the question about the method of interpretation – how is it possible for the present day humans to understand the action of the peoples long gone? We no longer have informants with whom researchers can enter into conversations or colleagues who can check the validity of our interpretation. In dealing with these issues, the age old issue about methods of interpretation – hermeneutic circle – needs to be revisited and considered.

## Conflict of Interest Statement

The author declares that the research was conducted in the absence of any commercial or financial relationships that could be construed as a potential conflict of interest.

## Footnotes

1 http://www.worldvaluessurvey.org/wvs.jsp

2
http://www.monitoringthefuture.org/

3
http://www.heri.ucla.edu/tfsPublications.php

4 http://ec.europa.eu/public_opinion/index_en.htm

5 http://www.afrobarometer.org/

6 http://www.arabbarometer.org/

7 http://www.asianbarometer.org/

8 http://www.vanderbilt.edu/lapop/about-americasbarometer.php

9 http://www.latinobarometro.org/lat.jsp

10 BP stands for Before Present. Typically determined by carbon dating, the “present” is defined as 1 January 1950, the start of the year in which the carbon dating method was established.
